# Bioconversion of α-pinene by a novel cold-adapted fungus *Chrysosporium pannorum*

**DOI:** 10.1007/s10295-014-1550-0

**Published:** 2014-12-09

**Authors:** Mariusz Trytek, Krzysztof Jędrzejewski, Jan Fiedurek

**Affiliations:** Department of Industrial Microbiology, Faculty of Biology and Biotechnology, Maria Curie-Skłodowska University, Akademicka St. 19, 20-033 Lublin, Poland

**Keywords:** Biotransformation, *Chrysosporium*, Pinene, Psychrotrophs, Terpenes, Verbenol, Verbenone

## Abstract

The psychrotrophic fungus *Chrysosporium pannorum* A-1 is reported for the first time as a novel biocatalyst for O_2_-promoted oxidation of α-pinene. GC–MS analysis indicated that the main products of the reaction were compounds of a high commercial value, verbenol (**1**) and verbenone (**2**). Exponentially growing cells (days 2–3) were about twice as active as cells in the late stationary phase in terms of the total concentration of products. The highest yields of **1** and **2** were obtained using three-day and two-day-old mycelia and a medium containing 1.5 and 1 % (v/v) of the substrate, respectively. The optimal time for the bioconversion of α-pinene varied from 1 to 3 days, and depended on the kind of product desired. Most of **1** was produced at a relatively high concentration of 360 mg/L after the first six hours of α-pinene bioconversion [with an average yield of 69 mg/(g _dry cell_ L aqueous phase)]. The oxidative activity of *C. pannorum* was identified across a wide temperature range of 5–25 °C, 10 °C being the optimum for the production of **1** and 20 °C for the production of **2**. Sequential addition of the substrate during 3 days of the biotransformation resulted in a significant increase in **1** and **2** up to 722 and 176 mg/L, respectively, and a 2-fold enhancement of product yield as compared to bioconversion with a single supply of α-pinene. The concentration of total conversion products in the culture medium reached 1.33 g/L [which corresponded product yield of 225 mg/(g _dry cell_ L)]. This represents probably the most promising result reported to date for oxidative biotransformation of α-pinene by a wild-type microorganism.

**Electronic supplementary material:**

The online version of this article (doi:10.1007/s10295-014-1550-0) contains supplementary material, which is available to authorized users.

## Introduction

Cold-adapted (psychrophilic and psychrotrophic) microorganisms have the ability to reproduce and grow at temperatures close to 0 °C. More than three-quarters of the earth’s surface is occupied by cold ecosystems, including ocean depths and polar and alpine regions. Although the cold habitats of the earth are teeming with life, there are few biotechnological applications using microbial psychrophiles at low temperatures [43]. Most of the previous studies have focused on biosynthesis of low-temperature-active enzymes (so-called psychrozymes) [4, 10, 16, 18].

Organisms native to cold environments achieve metabolic rates that are only slightly lower than those reached by organisms living at moderate temperatures. Due to their high enzymatic activities and catalytic efficiencies in the temperature range of 0–20 °C, cold-adapted microorganisms are particularly useful for biotransformation involving volatile compounds, such as flavors and fragrances subject to faster evaporation at higher temperatures thus making it possible to obtain high amounts of these valuable products. The first reaction that this group of microorganisms was found to play an effective role in was transformation of the volatile substrate *R*-(+)-limonene [45]. Recently, a microbial reaction system based on psychrotrophic *Mortierella minutissima*, which shows a high catalase activity, has been developed for the oxidation of this monoterpene, using hydrogen peroxide as the oxygenating agent in the culture [46]. Apart from the two studies mentioned, no further investigation has been performed on biotransformation of terpenes or other hydrophobic compounds with psychrophiles and psychrotrophs, which is especially striking in comparison with the number of studies on mesophiles. There are also few papers emphasizing the benefits of the bioprocesses carried out at low temperatures [10, 18, 21, 43]. One example is a study by Renniger et al. [37], in which *E. coli* was genetically engineered to be active at low temperatures in order to increase the biosynthesis of isoprenoids.

Oxidative transformation of abundant and low-priced monoterpenes, such as α-pinene and *R*-(+)-limonene, has a considerable potential for production of a wide variety of different terpenoid derivatives found in the plant kingdom. A vast number of oxygenated monoterpenes are the main flavor and fragrance impact molecules in essential oils with beneficial effects on health [33]. Since such natural products are present only at low levels in nature, it is not surprising that significant efforts have been directed toward finding alternative, synthetic sources of these remarkable compounds [6]. Selective oxidation of α-pinene with some biocatalysts is an important commercial reaction because it can yield valuable products, such as verbenone and verbenol, which are expensive (US$ 3,500/kg) flavoring compounds applied as components of pheromone traps.

Due to their pronounced camphor and mint flavor notes [48], they are also widely utilized in the food industry and are employed as intermediates in the synthesis of perfume, cosmetics as well as pharmaceuticals. A very important reason for the interest in verbenone as a desirable product is also that it can provide a possible chiral precursor for the asymmetric synthesis of the A-ring of the very effective anticancer diterpene, taxol^®^ [26, 50].

α-Pinene is a hydrophobic and volatile organic compound, which, as a main component of turpentine, a paper and pulp industry residue, is available in bulk quantities at a low price. Emitted from forest products industry processes, it gives rise to photochemical smog, which is why its fungal-mediated biodegradation in a gas-phase biofilter has been investigated as a method of treatment of α-pinene-polluted waste gases [22]. Many genera of bacteria, e.g., *Pseudomonas* [7, 12, 14, 30], and several fungal species [3, 24, 40, 47] have been reported as biocatalysts in the bioconversion of pinene, although the efficiency of most of them was limited by high volatilization and toxicity of the substrate. This resulted in bioconversion yields in the range of several milligrams per liter, which were rather low compared to other terpene biooxidations. Promising results have been published recently by Schewe et al. [42], who obtained total products (pinene oxide, verbenol and myrtenol) of over 1 g/L using a recombinant *Escherichia coli* in an aqueous-organic two-phase system. Active and stable biocatalysts, resistant to toxic monoterpenes are still being searched for [38].

Until now, no data have been published either on the ability of psychrotrophic fungi to biocatalyze oxidation of pinenes or on the biotransformation of this kind of precursors at temperatures below 25 °C. Therefore, the aim of the present work was to test the biotechnological capability of the fungus *Chrysosporium pannorum* to catalyze an oxidation reaction at low temperatures, as well as to investigate the best operational conditions for reaching a high biocatalysis yield of high-value terpenoids from α-pinene.

## Materials and methods

### Chemicals

(1*S*)-(−)-α-Pinene (98 %), (*S*)-*cis*-Verbenol (95 %), and dodecane (99 %) were purchased from Sigma-Aldrich, USA. (−)-Linalool (>97 %) and (1*S*)-(−)-verbenone at a purity of >97 % were obtained from Fluka, Switzerland and stored at 5 °C.

### Fungus and media

The psychrotrophic fungus *Ch. pannorum* A-1 used in this study had been isolated from soils collected in the Arctic tundra (West Spitsbergen). The microorganism was maintained on malt agar slants, stored at 3 °C, and subcultured every month. Cultivation and bioconversion experiments were conducted in a liquid basal medium (BM) consisting of malt extract 1 %, peptone 0.5 %, glucose 1 %, and yeast extract 0.5 %.

### Batch operation

After 6 days of growth on agar slants at 20 °C, spores of *Ch. pannorum* A-1 were harvested, washed twice with sterile 0.1 M McIlvaine buffer, pH 5.0, and filtered through glass wool to remove hyphal fragments. Concentration of spores was adjusted to 2 × 10^5^ mL^−1^.

Erlenmeyer flasks (100 mL) containing 25 mL of the medium were sealed with cellulose plugs and autoclaved for 20 min at 121 °C. After cooling, the media were inoculated uniformly with 2 ml of fungal spore suspensions (containing about 4 × 10^5^ spores) and cultivated at 20 °C on a rotary shaker (150 rpm).

After the specified time of fungal growth, bioconversion experiments were started by just adding α-pinene to the culture (containing pre-grown mycelium). The amount of substrate added to the medium depended on the specific experiment and ranged from 0.3 to 1.5 % (v/v). The time of substrate addition to the mycelial culture also differed between experiments and was correlated with the age of the mycelium.

All α-pinene biotransformations were carried out in parallel with controls, in exactly the same conditions using the heat-inactivated microorganism which had been autoclaved at 121 °C for 15 min.

To determine the growth curve, two flasks with total broth (without α-pinene) were periodically taken at the following time intervals: 0, 6, 14, 24, 38, 48, 62, 72, 86, 96, and 110 h. Growth was followed by measuring the dry weight of mycelium. For the kinetic experiment, two flasks (as independent samples) were sacrificed at each sampling time after 2, 6, and 12 h and then every 12 h up to 108 h, to assay the products of biotransformation.

### Biotransformation analysis

After a specified time of biotransformation, 500 µL of a 0.1 % internal standards (IS) solution in hexane was added to the medium. The biomass was harvested by filtration, and the liquid for product recovery was extracted twice by an equal volume of diethyl ether in a separatory funnel. The ether fraction was separated, dried over anhydrous sodium sulfate, and concentrated to dryness on rotary vacuum evaporators at a water bath temperature of 40 °C. The residues obtained were dissolved in 4 mL of hexane and used for GC and GC–MS analyses conducted according to the method reported previously [46].

Volatile compounds were quantified by comparing with the internal standard (IS) added, using a calibration curve of peak area ratios (analyte/IS) vs. amount ratios (analyte/IS) from standard authentic samples. 0.1 % solutions (w/v) of *n*-decane (for the substrate) and linalool (for oxidation products) in hexane were used as internal standards for gas chromatography. Terpenoids were identified by fitting their mass spectra to those from the NIST 2004 and MassFinder 3 library and by an additional comparison of the GC retention indexes of standard compounds.

Biotransformations were performed in two replicate samples, and the analyses were carried out in duplicate. The error associated with the GC quantification of the samples was ±6 % and is quoted for a confidence interval of 94 %. The data presented are reported as average values.

### Respirometric measurements

After a predetermined time of growth (1, 2, 3, 4 or 5 days) at 20 °C, 50-mL mycelial culture (obtained from two Erlenmeyer flasks) of *C. pannorum* containing a fixed amount of fungal mycelium (about 5 g of dry mass per L) was aseptically transferred into a 250-mL reactor (6.5 × 18.0 cm) with an electrode placed at the bottom. Finally, the samples were aerated to 100 % of dissolved oxygen (DO) concentration, delivered with 1.5 % (v/v) of α-pinene, sealed from atmospheric oxygen and incubated with magnetic stirring (150 rpm) under a controlled temperature (20 °C). The rate of oxygen uptake by the fungus was measured using an Ingold electrode (Mettler-Toledo Inc., Columbus, OH, USA). The electrode was calibrated at the beginning of each experiment by measuring its signal in the air-saturated media before adding the mycelium. The values of the readings were expressed as percentage of the initial DO level.

All experiments were carried out by direct sampling from the pellet culture, always attempting to obtain representative samples for the different pellet sizes and mycelium weights.

## Results and discussion

**Fig. 1:**
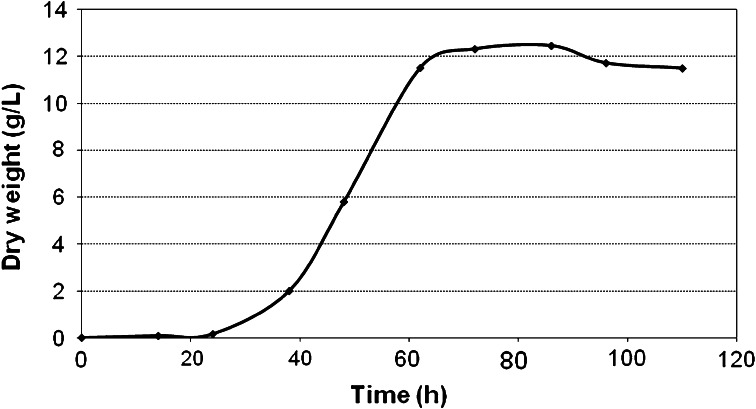
Growth curve for the psychrotrophic fungus *Ch. pannorum* A1 growing in a liquid medium (without α-pinene) at 20 °C

The fungus *Ch. pannorum* A-1 was chosen for the study from among a group of psychrotrophic microorganisms showing good activity in the biotransformation of *R*-(+)-limonene [45]. A preliminary investigation had demonstrated that the most beneficial conditions for growing small pellets of *Ch. pannorum* A-1 were: the agitation speed of 150 rpm, culture basal medium BM, and a temperature of 20 °C, which was the growth optimum (data not shown). In these conditions, the mycelial pellets reached sizes ranging from 1 to 2 mm. A small size of fungal pellets ensures better mass transfer of the hydrophobic substrates and oxygen to the biocatalyst, which is a very important determinant affecting the bioconversion rate. The dynamics of mycelial growth in the liquid medium at 20 °C (in the absence of monoterpene), as graphically depicted in Fig. 1, reveal the three phases normally found in physiological characteristics and show that the fungus grew at a high rate, reaching the stationary phase already after 60 h of growth. The lag phase in this case was about 24 h, and growth declined after about 84 h.

**Fig. 2:**
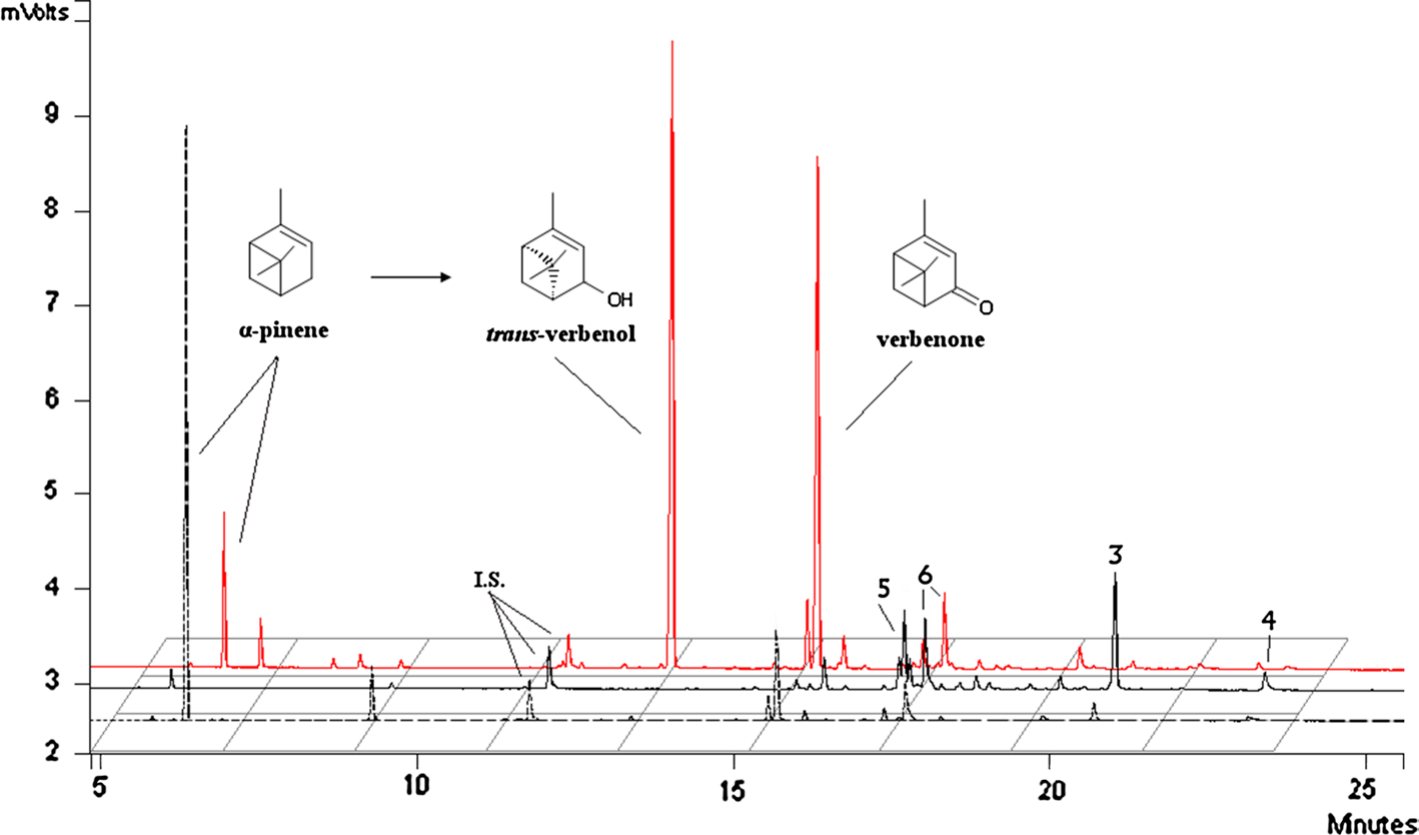
Main products and GC-FID chromatograms of the post-reaction mixtures after 48 h (further chromatogram) and 96 h (closer chromatogram) of α-pinene biotransformation by *Ch. pannorum* A1 at 0.5 % (v/v) of initial substrate concentration. 3–6 are unidentified compounds. The chromatogram with a *dashed line* represents control samples [0.5 % (v/v) α-pinene, 48 h] with the heat-inactivated microorganism

GC–MS analysis of the products obtained in the first biocatalytic experiment after 2 days of biotransformation at [0.5 % (v/v) α-pinene] indicated that allylic hydroxylation of α-pinene in its C-3-position was the most characteristic reaction for the *C. pannorum* species. The main products were compounds of a high commercial value, verbenol (**1**) and verbenone (**2**) (Fig. 2).

Prolonged biotransformation, especially at a low initial substrate concentration (0.3–0.5 %), also generated considerable amounts of four new unidentified compounds (3–6), as shown on typical chromatograms (Fig. 2). Their mass spectra are shown in Supplement 1.

Verbenol and verbenone have previously been obtained by biotransformation of α-pinene with fungi [3, 24, 47, 48] and bacteria [14, 39, 42], but in yields that were insufficient for industrial-scale production. In nature, these terpenoids are active components of *Verbena triphylla* plants, hyssop and rosemary oils, and are also known as pheromones of many insects and tree pests such as bark beetles. The (+) form of verbenol is known as the aggregation pheromone for the species *Dendroctonus*, while the (−) form of verbenol is an intermediate in the synthesis of terpadienes and the aggregation pheromone for the spruce bark beetle *Ips typographus* [17, 27, 34]. (−)-Verbenone has been found to be a flavor constituent of strawberry, raspberry, dill, rosemary and spearmint flavor mixtures [36].

**Fig. 3:**
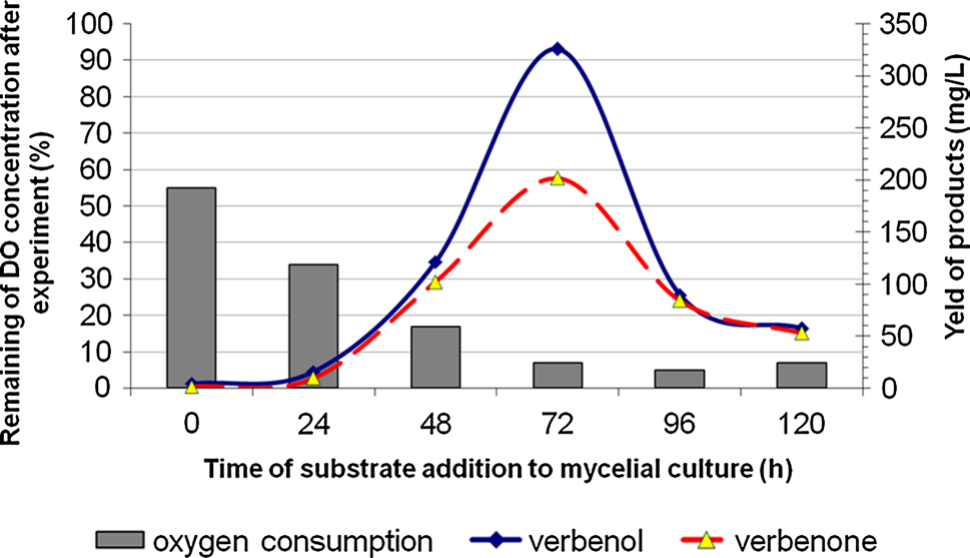
Relationship between the efficacy of α-pinene bioconversion and the rate of oxygen consumption by the fungus *C. pannorum* as a function of age of mycelium. Bioconversion conditions: temperature, 20 °C; time 72 h; initial α-pinene concentration, 1.5 % (v/v); medium volume, 25 mL. Oxygen uptake was expressed as percentage of the initial level of saturation DO (after 10 min of incubation)

In further experiments, the effect of substrate concentration and the time of its addition to the fungal culture (i.e. the age of the biomass) on biocatalytic efficiency of *Ch. pannorum* A-1 were studied in order to counteract the detrimental effects of terpene toxicity toward the mycelium. Oxygen consumption rates, in the presence of a relatively high, 1.5 % (v/v), fraction of α-pinene, depending on the age of the mycelium were also investigated. The oxygen uptake rate can serve here as an additional parameter indicating the relative metabolic activity of living mycelia; the higher the value of this parameter, the higher the activity of the fungus. The highest biotransformation yield (at 1.5 % of α-pinene) was found to be positively correlated with the most rapid oxygen consumption by the fungus, and this was observed when α-pinene was added directly to the cultures with mycelium that had been pre-grown for 72 h, i.e., at the beginning of the stationary phase (Fig. 3).

**Fig. 4:**
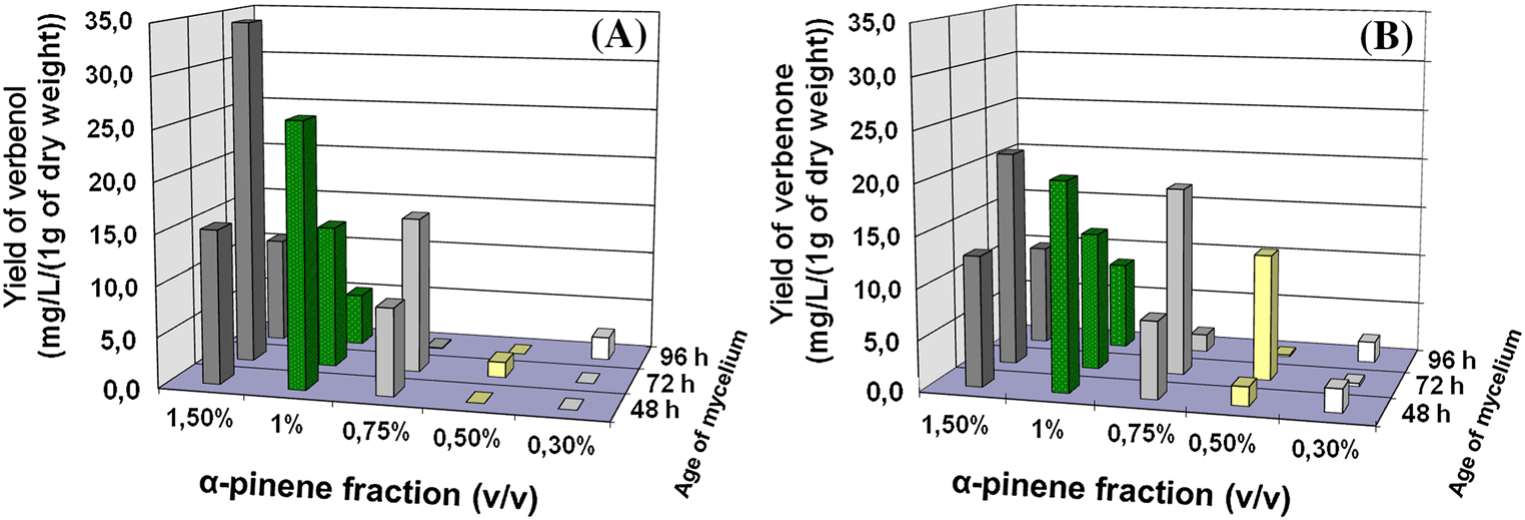
Effect of α-pinene concentration and time of its addition to the culture medium on the production of verbenol (**a**) and verbenone (**b**) in submerged culture of *Ch. pannorum* A-1. Biotransformation time: 72 h

Such results may point to the higher resistance of the fungus to toxic terpenes at this physiological stage. A further study proved that exponentially growing cells (days 2–3) were about twice as active as cells in the late stationary phase (96 h) in terms of the total concentration of biotransformation products (Fig. 4). This finding is consistent with data concerning limonene biotransformation by the mesophilic *Penicillium* sp., the biocatalytic activity of which was significantly enhanced in the log phase of growth [44]. Also several previous studies on whole-cell oxygenations have revealed that cells reaching the stationary phase after batch or fed-batch growth steadily loose their oxygenase activities despite the supply of an energy source [8, 9, 15, 20, 28, 29, 49]. Such an effect may be connected with the decreasing level of intracellular oxygenase and cofactor deficiency resulting from a reduced NADH regeneration rate when the cells adapt their metabolic activity to the actual growth stage. In our earlier experiments, carried out in oxygen-limited growth conditions, we obtained significantly lower amounts of the products than those normally obtained in aerobic conditions (data not shown).

The highest yield of **1** (325.8 mg/L), under the tested conditions, was achieved using 72 h-old mycelium and a medium containing 1.5 % (v/v) of the substrate (Fig. 4). Somewhat higher concentration of the product **2** (203.4 mg/L) was obtained for the combination of 1 % (v/v) α-pinene and 48 h-old mycelium. A sharp drop in the bioconversion yield, on the other hand, was observed for 24 h-old mycelium, when only a trace amount of products was obtained. This can be explained by the extreme sensitivity of young mycelia to terpene toxicity.

**Fig. 5:**
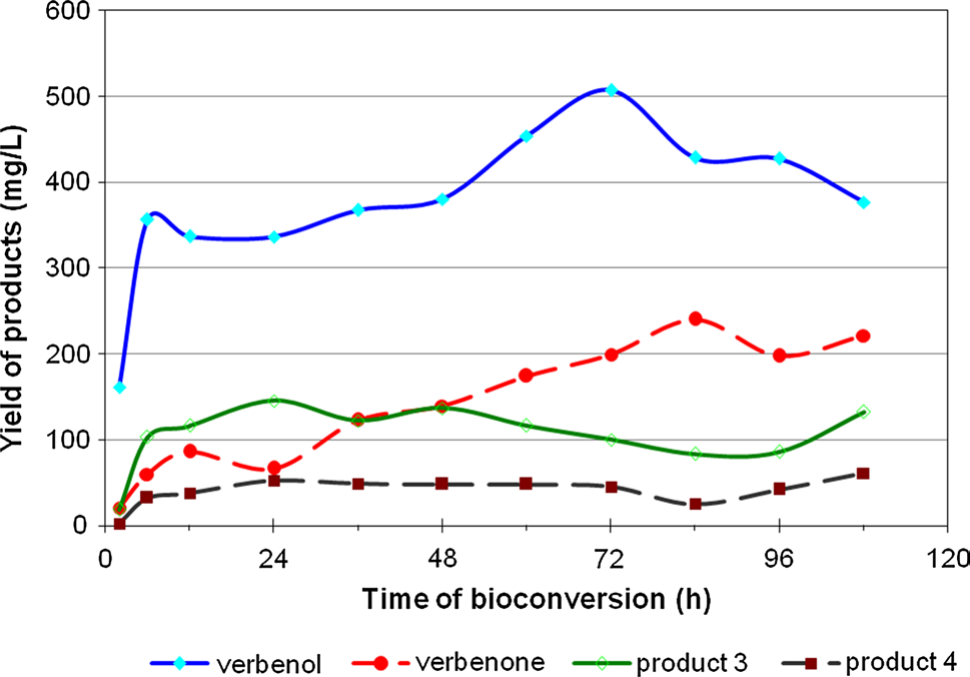
Time course of α-pinene biotransformation by *Ch. pannorum* A1

Time course studies demonstrated that the optimal time for the bioconversion of α-pinene varied from 1 to 3 days, and depended on the kind of product desired (Fig. 5). Most of **1** was produced at a relatively high concentration of 360 mg/L after the first six hours of α-pinene bioconversion [with an average product yield of 69 mg/(g _dry cell_ L aqueous phase)], whereas the concentration of **2** grew from the 24th hour of the biotransformation on, reaching a maximum after 84 h.

Presumably, the extended time of verbenone production in the present study was caused by a gradual release of trapped products to the medium from the membrane cell, preceded by partial overoxidation of verbenol. A similar conversion time was observed by Bhattacharyya et al. [5, 35], in their pioneering studies on biotransformation of α-pinene to verbenol, verbenone, and *trans*-sobrerol by *Aspergillus niger*, and in later works on this fungal strain [3] as well as on *Penicillium digitatum* strains used for biotransformation of limonene [2, 13, 44]. In the case of bacterial biocatalysts, such as recombinant *E. coli* BL21 and *Pseudomonas* sp., optimal α-pinene bioconversion was obtained after more than 4 and 48 h, respectively [42, 51]. In turn, the time of α-pinene oxidation by *C. pannorum* (1–3 days) seems to be shorter than that of monoterpene conversion performed with the mesophilic molds *A. niger* [38, 40] and *A. cellulosae* [31], which was reported to be 7 days. Longer bioconversion times were also observed for the yeasts *Candida tropicalis* (12 days) [11] and *Hormonema* sp. (7 days) [47], and the basidiomycete *Pleurotus sapidus* (12 days) [32].

**Fig. 6:**
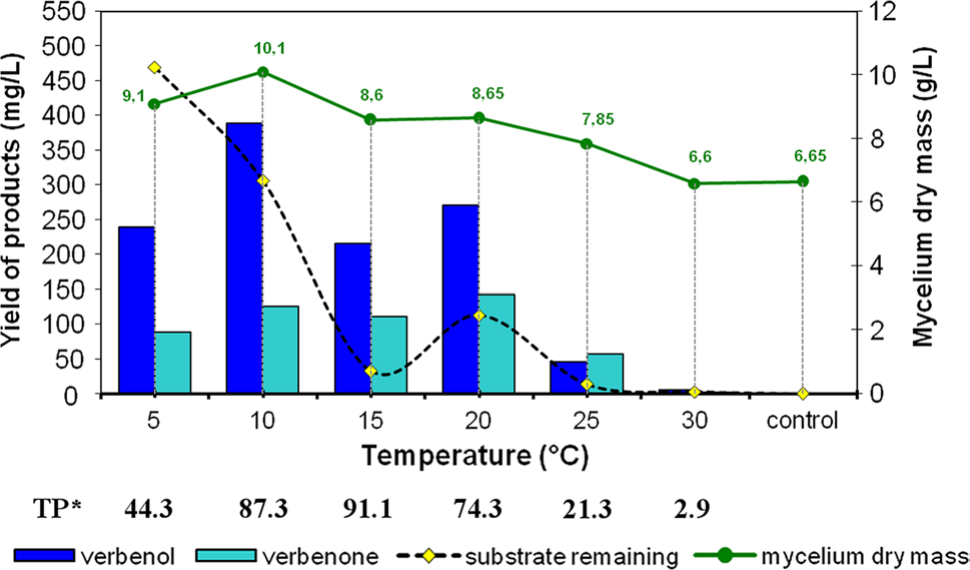
Effect of temperature on fungal growth and efficiency of α-pinene biotransformation in submerged culture of *Ch. pannorum* A-1

In a subsequent study, the biooxidation activity of *C. pannorum* was indentified across a wide temperature range of 5–25 °C, 10 °C being the optimum for the production of **1** and 20 °C for the production of **2** (Fig. 6). The relatively high catalytic activity at 5 °C and its dramatic drop at 30 °C confirmed that the fungus examined belongs to cold-adapted microorganisms. The optimum temperature for the catalytic activity of *Ch. pannorum* A-1 was 15 °C. It was in these conditions that the highest substrate depletion was noted, and prominent peaks of unknown products (3–6) appeared in the chromatogram of the post-reaction mixture (data not shown). It was also found that at 15 °C, as compared to 5 and 10 °C, there was a decline in fungal growth during biotransformation, which increased product yield [TP^*^ expressed in mg of total products/(g _dry cell_ L aqueous phase)].

This effect is suggested to be a result of inhibition various cellular reactions in living mycelia, especially oxidative phosphorylation, which are considered to compete with the biotransformation pathway for NAD(P)H needed for the reductive activation of molecular oxygen [25, 41]. It is worth noting that the mycelial biomass of *Ch. pannorum* A-1 in the cultures with the addition of α-pinene was higher than in control probes without pinene (performed at 20 °C), which points to partial utilization of α-pinene as an energy source in the process of biomass formation. When the temperature was increased to 30 °C, both growth and bioconversion efficiency were reduced due to the intrinsic physiological properties of psychrotrophs.

It has been found that bioprocesses carried out at low temperatures have several advantages. For example, the production of isoprenoids has been shown to be increased by lowering the temperature below the temperature that supports the maximum specific growth rate [37]. Although the system studied was somewhat different from our biocatalytic system, the use of microorganisms capable of transforming various organic precursors at low temperatures seems to be an attractive alternative to the traditional mesophilic-based biotechnological processes, especially those involving easily volatilized substrates and products. It must also be remembered that the average temperature of the Earth’s surface is about 15–17 °C [23]. The cold-adapted biocatalyst examined in this present study would be able to transform over a wide range of seasonal temperature fluctuations, thus offering economic benefits through energy savings. To our knowledge, the lowest temperatures at which biotransformation of monoterpenes has been performed were 24 °C for *Pleurotus sapidus* [32] and 25 °C for *Hormonema* sp [47], both characterized by a longer bioconversion time as compared to *Ch. pannorum* A-1. In this context, the investigated psychrotrophic fungus (beside *Mortierella minutissima* 01) is, so far, the only biocatalyst reported to be suitable for terpene biotransformation at lower ambient temperatures; it is also one that may, thanks to its good catalytic performance, contribute to the development of an environmentally friendly strategy for production of valuable compounds, especially at cold seasons and in cool climate regions.

**Fig. 7:**
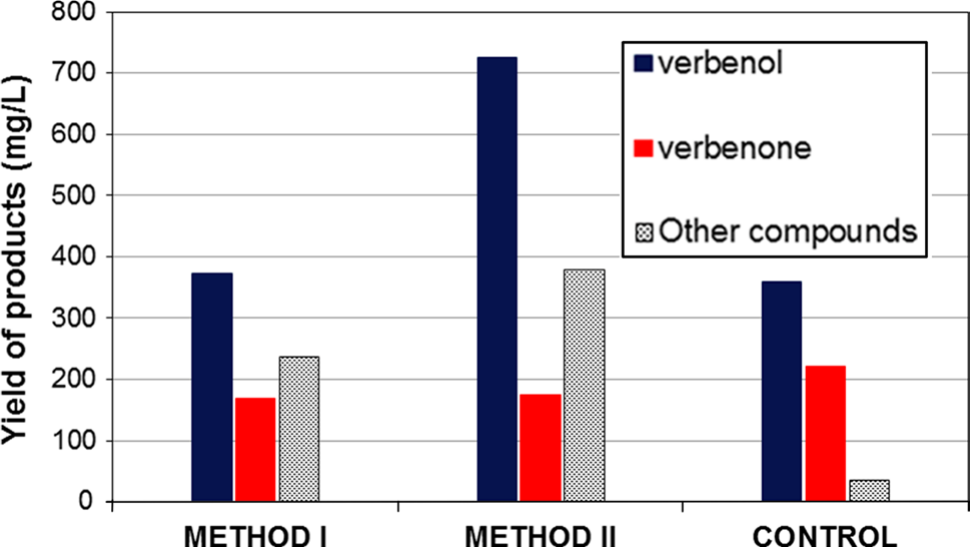
Effect of sequential addition of α-pinene to the culture medium on total product yield *Method I* 0.3 % (v/v) fraction of α-pinene was added to the reaction medium containing two-day old-mycelium and then in doses of 0.5 and 0.7 % (v/v) after 6 and 12 h, respectively, after which bioconversion was continued for 36 h. Total bioconversion time: 48 h. *Method II* 0.2 % (v/v) fraction of α-pinene was added to the reaction medium containing one-day-old mycelium and then in doses of 0.3, 0.5 and 0.5 % (v/v) after every 12 h, after which bioconversion was continued for 36 h. Total bioconversion time: 72 h; temperature 20 °C; the biomass weight after biotransformation using method I and II was 8.8 and 5.9 g/L, respectively. *Control* 1.5 % (v/v) of α-pinene was added as single dosage to reaction medium containing 72 h-old mycelium. Time of bioconversion: 72 h

When a biotransformation is limited by substrate availability, whether due to the hydrophobic character or toxicity of the substrate, its slow addition into the reaction medium is a common solution [19]. Substrate-limiting feeding keeps substrate concentrations below inhibiting levels, which, in the case of bioconversion of low-aqueous-soluble monoterpenes, allows the attainment of high yields of terpenoids [1, 44]. Tan et al. [44], for instance, reported a 12-fold increase in the activity of the microorganism they studied when sequential substrate feeding was used in the biooxidation of limonene to *R*-(+)-α-terpineol. We compared two methods of sequential α-pinene addition (Fig. 7). The use of method **II** yielded nearly 1.33 mg/L of total products [corresponding to the product yield of 225 mg/(g dry cell wt L)] as compared to 912 mg/L obtained using method **I**. The gradual addition of the substrate during 3 days of the biotransformation resulted in a significant increase in verbenol and verbenone of up to 722 and 176 mg/L, respectively, and a 3-fold increase in productivity as compared to the bioconversion with a single supply of α-pinene (Fig. 7).

The enhancement of the transformation yield was most probably a result of the adaptation of fungal metabolism to and/or its induction by the increasing amount of the substrate as well as the reduction of its cytotoxic effect at high fractions. The specific yield achieved was estimated to be about 20 times higher than that determined for the mesophilic bacteria *Pseudomonas sp.* [51]. These results are promising in the context of future studies on adding the substrate at different concentrations at the various steps of fungal cultivation, especially in connection with the induction of cell enzymes by terpenoid substrates as well as the use of hydrophilic co-solvents.

## Conclusions

*Ch. pannorum* A-1 was shown to be a very active catalyst for O_2_-promoted oxidation of α-pinene. Oxidation of α-pinene by the cold-adapted fungus *C. pannorum* leads to the generation of natural flavor and fragrance derivatives (verbenone and verbenol). The fact that *Ch. pannorum* A-1 is the most active in the temperature range from 10 to 20 °C means that it can be employed in biotransformation processes at a wide variety of ambient temperatures, thus reducing energy consumption. Gradual addition of the substrate during 3 days of the biotransformation at a low temperature resulted in a significant increase in terpenoid productivity. The concentration of total conversion terpenoids in the culture medium reached 1.33 g/L, making the fungus one of the most productive biocatalysts of α-pinene oxidation. The highest yield of verbenol, under the conditions tested, was obtained using 72 h-old mycelium and a medium containing 1.5 % (v/v) of the substrate at 10 °C, whereas the largest amount of verbenone was obtained using 48 h-old mycelium and a medium containing 1.0 % (v/v) of the substrate at 20 °C. This may indicate that different biotranformation products require different process parameters to be produced at high yields This result also promises future improvement of terpenoid productivity, which can be achieved both by the use of cell immobilization and by performing bioprocesses in aqueous-organic biphasic systems using biocompatible organic solvents. The use of continuous, slow substrate feeding and in situ product recovery may also help overcome toxicity- and solubility-related limitations.

## Electronic supplementary material

Below is the link to the electronic supplementary material.

Supplementary material 1 (DOC 31 kb)
