# Supplementary material for: Bioconversion of α-pinene by a novel cold-adapted fungus Chrysosporium pannorum
Source: J Ind Microbiol Biotechnol. 2014 Dec 9;42(2):181–8. doi: 10.1007/s10295-014-1550-0 (PMC4293472; doi:10.1007/s10295-014-1550-0)
Supplement: Supplementary file 1 — Supplementary material 1 (DOC 31 kb) [file 10295_2014_1550_MOESM1_ESM.doc]

Supplement 1. Mass spectra of the identified unknown monoterpene products of α-pinene biotransformation by *Chrysosporium pannorum* A-1.

Compound **5**

Compound **6**

Compound **3**

Compound **4**
